# Prevalence of rheumatoid arthritis and diagnostic validity of a prediction score, in patients visiting orthropedic clinics in the Madinah region of Saudi Arabia: a retrospective cross-sectional study

**DOI:** 10.7717/peerj.14362

**Published:** 2022-11-15

**Authors:** Shabir Ahmad Mir, Mamdooh Noor, Md Dilshad Manzar, Bader Alshehri, Mohammed Alaidarous, Abdul Aziz Bin Dukhyil, Saeed Banawas, Yahya Madkhali, Sadaf Jahan, Faizan Z. Kashoo, Danish Iqbal, Qamar Zia, Suliman A. Alsagaby, Sahar ALDosari

**Affiliations:** 1 Department of Medical Laboratory Science, College of Applied Medical Science, Majmaah University, Al Majmaah, Riyadh, Saudi Arabia; 2 Department of Nursing, College of Applied Medical Science, Majmaah University, Al Majmaah, Riyadh, Saudi Arabia; 3 Health and Basic Sciences Research Center, Majmaah University, Al Majmaah, Riyadh, Saudi Arabia; 4 Department of Biomedical Sciences, Oregon State University, Corvallis, Oregon, United States; 5 Department of Physical Therapy and Rehabilitation, College of Applied Medical Science, Majmaah University, Al Majmaah, Riyadh, Saudi Arabia

**Keywords:** Clinical presentation, Co-morbidity, Disease marker, Diagnosis, Rheumatoid arthritis

## Abstract

**Introduction:**

In Saudi Arabia, the epidemiology of rheumatoid arthritis (RA) is not well studied and is marked by inconsistencies in clinical diagnosis. Therefore, in this study, we explored the prevalence, clinical characteristics, and diagnostic validity of a prediction score based upon disease markers in orthropedic clinics’ patients in the Madinah region of Saudi Arabia.

**Method:**

The clinical data for this retrospective cross-sectional study were retrieved from the database registry of orthopedic clinics in selected hospitals of the Medinah province of Saudi Arabia. Sociodemographic features, disease markers and the clinical characteristics were collected for a period of 6 months, from December 1, 2020, to May 31, 2021. The prediction score was generated from the sum of disease markers, coded as dichotomous variables.

**Results:**

The total sample size of our study was 401. The prevalence of RA in the study subjects (*n* = 401) was 14.46% (*n* = 58). Among RA patients, the majority were females (60.3%). Painful joints (69%) and swollen joints (51.7%) were the most common clinical complaints among RA patients. RA patients suffered from arthritis (51.7%) and experienced fatigue (46.6%), weight loss (44.8%), and loss of appetite (41.4%). Diabetes (55.2%) was the most common comorbidity in the RA patients. The sensitivity and specificity of the prediction score at the criterion score of 2.5 were 67.3% and 63.0%, respectively. The area under the curve was 0.69 (95% CI [0.62–0.76]).

**Conclusion:**

There was a moderately high prevalence of RA in patients visiting the orthropedic clinics of the selected hospitals of Madinah region of Saudi Arabia. The diagnostic validity of the prediction score, though promising, was slightly lower than the acceptable range.

## Introduction

Rheumatoid arthritis (RA) is a severe inflammatory illness that mainly affects middle-aged people and destroys tissues located in the synovial joints such as those of hands and knees. RA affects about 0.5–1.1% of the global population (Al-Dalaan et al., 1998; Al-Ghamdi, 2009; Albishri, Alsabban & Altwairqi, 2015). The development of the disease increases with age, and it is more prevalent in females, especially in developed countries (Almoallim & Alharbi, 2014; Almoallim et al., 2020). The main symptoms of RA include swelling, stiffness, and pain in the joints. However, it is difficult to identify the disease in its early stages because its manifestations are similar to those of other conditions (American College of Rheumatology Subcommittee on Rheumatoid Arthritis Guidelines, 2002). Furthermore, the treatment of RA is difficult because the disease responds differently to various types of interventions (Anderson et al., 2012). Delayed treatment of RA usually results in permanent disabilities, reduced quality of life, and in severe cases, death (Badsha, Kong & Tak, 2008). RA patients are 50% more likely to experience premature death compared to the other population members (Bruce & Fries, 2003). Currently, medical experts employ various methods to diagnose RA and assess the therapeutic results. These methods include direct assessments and more advanced methods, including imaging and ultrasound (United Rheumatology, 2016).

RA affects almost all parts of the world, however, studies have shown that the prevalence of the disease varies among different ethnicities and geographic regions (Costenbader et al., 2008; Cross et al., 2014; Dargham et al., 2019). The prevalence rate of the disease in developing countries is generally low compared to the rates in the developed countries. Age, socioeconomic status, and ethnicity are considered as the risk factors that influence the development of RA (Deane et al., 2017; Dessie et al., 2021). The epidemiology and prevalence of RA in the Middle East and North Africa (MENA) region is not clearly understood. Only a limited number of studies have investigated the incidence and prevalence rates of RA among the MENA region population (Dessie et al., 2021; Favalli et al., 2019; Felson et al., 2011; Gabriel & Michaud, 2009; Guo et al., 2018; Halabi et al., 2015). A study conducted on the spectrum of rheumatic diseases in Saudi Arabia revealed that RA was the most prevalent inflammatory arthritis in the region and was less severe than that experienced in the developed countries (Heegaard et al., 2013). It is essential to investigate the prevalence rates of the disease in Saudi Arabia because the extent of the problem in the whole population is not clear. Therefore, in the present study we aimed to investigate the prevalence and the clinical characteristics of RA in Madinah region of Saudi Arabia.

Evidence shows that there is a lack of adequate sensitivity and specificity in clinical diagnosis of RA (Hirata et al., 2015). This suggests that in spite of the existing expert diagnostic criteria (Hirata et al., 2015; Hosmer & Lemeshow, 2000; Houge et al., 2020), RA diagnosis is clinically challenging because of its multiple symptoms and clinical presentations that resemble with those of other similar diseases. Therefore, continuous and concerted research efforts are necessary to explore new efficient strategies. In this regard, we explored development of a prediction score based upon the disease activity markers to assist in the diagnosis process.

## Methodology

### Study site and design

This retrospective study was conducted in Medinah province, which is located in the western region of Saudi Arabia. The population of the Madinah region was estimated to be approximately two million in 2018. In this study, retrospective clinical data was collected for a period of six months, from December 1, 2020 to May 31, 2021. The study included patients above 16 years of age, who visited the orthropedic clinics of Madinah General Hospital (MGH), King Fahad Hospital (KFH) and Ohud Hospital (OH) of Madinah. The three hospitals were purposively selected in Medinah. The choice of the city was selected on convenience criteria. Information of all the patients who met the inclusion criteria were extracted. The patients below 16 years of age and those with incomplete entries were excluded. The sociodemographic and the clinical data of the patients was collected from the database registry of MGH, KFH and OH and the collected data was used to analyze the sociodemographic features and the clinical characteristics of the patients included in this study. The sample size in this study was 401 participants which is more than the minimum sample required of 351 as determined by the method described by Jones, Carley & Harrison (2003) for diagnostic studies using these assumptions: minimum specificity of 80%, 5% confidence interval, 30% for expected prevalence of the target condition (Hussain et al., 2016). Clinical data of all the patients registered in the data registry of the ortho clinics of selected hospitals from December 1, 2020 to May 31, 2021, was collected and analysed in this study.

As the study used a retrospective design, informed written consent from individual participants was not obtained. This study design reviewed and approved by the Institutional Review Board (IRB), General Directorate of Health Affairs in Madinah (IRB Registration Number H-03-M-084).

### Clinical diagnosis of RA

Basic and well-established serological tests including the rheumatoid factor (RF) and C-reactive protein (CRP) tests, and erythrocyte sedimentation rate (ESR), a hematological test, were performed for laboratory diagnosis of RA in the patients visiting orthopedic outpatient clinics of the selected hospitals in Madinah region. The patients with ESR >28 mm/h were positive for ESR, and those with CRP level >8 mg/L, and RF ≥20 IU/mL had been considered positive for CRP and RF, respectively. In addition, other clinical characteristics (arthritis, fatigue, weight loss, loss of appetite, fever, arthralgia, Raynaud syndrome, myalgias, *etc.*), and other markers of the disease (painful joint count, swollen joint count, erosive arthritis, *etc.*) were also considered by the clinician during the diagnosis of the patients. The criteria from the American College of Rheumatology (ACR) were applied by the clinician during the diagnosis of RA (Hirata et al., 2015; Hosmer & Lemeshow, 2000; Houge et al., 2020) and the final diagnoses of the patients as confirmed by the expert clinicians in the selected orthopedic clinics was revealed from their clinical records.

### Prediction score of RA disease markers

The prediction score was generated by the algebraic sum of the disease marker scores coded as dichotomous variables (‘0’ for absence, and ‘1’ for presence of the markers). The disease markers whose dichotomous scores were added were: presence of painful joint count, presence of swollen joint count, presence of high ESR (>28 mm/h), positive for CRP level (>8 mg/L), positive for rheumatoid factor (≥20 IU/mL), and presence of erosive arthritis.

### Statistical analysis

The collected data was analyzed using the Statistical Package for Social Sciences (SPSS) for Windows, version 23 (SPSS Inc., Chicago, IL, USA). The continuous variables were described using means and standard deviations, whereas, the categorical variables were described by frequencies and percentages. All analyses were descriptive; without any formal statistical comparisons. A receiver operating characteristic curve (ROC) analysis was performed to determine the diagnostic accuracy of a prediction score (test variable) with respect to the clinical diagnosis (state variable).

## Results

### Sociodemographic characteristics of the study subjects

The main purpose of the present study was to identify the prevalence and clinical characteristics of RA in patients visiting the orthopaedic clinics of the selected hospitals of Madinah province located in the western region of Saudi Arabia. This study included the clinical data of 401 patients who visited the orthopedic clinics of MGH, KFH, and OH of Madinah from December 1, 2020, to May 31, 2021. The mean age of the included patients was 48.75 ± 15.83 years. Out of the 401 patients, 104 (25.9%) were males whereas the remaining 297 (74.1%) were females. Most of the patients (89.3%) involved in this study were Saudi citizens with only 10.7% patients being non-Saudi residents. Based upon their final diagnosis the patients were divided into two categories: RA patients and non-rheumatoid arthritis (NRA) patients. The various sociodemographic characteristics considered in this study and their distribution in the study population and the categories thereof (RA and NRA) have been summarized in Table 1. The prevalence of RA in patients visiting the orthopaedic clinics of the selected hospitals of Madinah region as estimated in the current study was found to be 14.46%. Our results revealed that 60.3% of the RA patients were female, whereas, 39.7% of the RA patients in this study were men (Table 1), supporting the available information that women are more likely to be affected by RA than men.

**Table 1:** Sociodemographic characteristics of the study subjects (patients who visited the orthopedic clinics of the selected hospitals in Madinah city from December 2020 to May 2021).

Characteristics	All participiant patients (*n* = 401) mean ± SD/frequency (percentage)	Non-Rheumaoid arthritis patients visiting orthopedic clinic (*n* = 343) mean ± SD/frequency (percentage)	Rheumatoid arthritis* patients (*n* = 58) mean ± SD/frequency (percentage)
Age (year)	48.75 ± 15.83	48.55 ± 15.39	49.97 ± 18.34
Gender Male Female	104 (25.9) 297 (74.1)	81 (23.6) 262 (76.4)	23 (39.7) 35 (60.3)
Nationality Saudi Non-Saudi	358 (89.3) 43 (10.7)	306 (89.2) 37 (10.8)	52 (89.7) 6 (10.3)
Marital status Single Married Divorced Widowed	77 (19.2) 237 (59.1) 38 (9.5) 49 (12.2)	66 (19.2) 206 (60.1) 32 (9.3) 39 (11.4)	11 (19) 31 (53.4) 6 (10.3) 10 (17.2)
Residential area Urban Rural	355 (88.5) 46 (11.5)	305 (88.9) 38 (11.1)	50 (86.2) 8 (13.8)
Occupation Working Non-working Household Chores	151 (37.7) 156 (38.9) 94 (23.4)	134 (39.1) 128 (37.3) 81 (23.7)	17 (29.3) 28 (48.3) 13(22.4)
Habit Smoking Non-smoking	66 (16.5) 335 (835)	53 (15.5) 290 (84.5)	13 (22.4) 45 (77.6)

**Note:**

* Based on clinical expert’s diagnosis.

### Markers of disease activity, clinical presentations and co-morbidities of RA patients

The clinical data for the important markers of the RA disease activity including painful joint count, swollen joint count, erythrocyte sedimentation rate (ESR), C-reactive protein (CRP), rheumatoid factor (RF), and erosive arthritis was collected and analyzed in this study. The majority of the RA patients were positive for four disease activity markers, *i.e*., painful joints, swollen joints, ESR (>28 mm/h), and RF (≥20 IU/mL) (Table 2). Most of the RA patients (91.4%) were simultaneously positive for two or more of the disease markers. The blood profile of the patients with RA showed increased erythrocyte sedimentation rate and CRP. Prompt treatment is needed among patients showing increased disease markers as untreated patients become work-disabled within 2 to 3 years of diagnosis.

**Table 2:** Markers of disease activity in rheumatoid arthritis patients included in this study.

Disease activity markers	Rheumatoid arthritis* patients (*n* = 58) Frequency (percentage) of those recording presence of the disease activity markers
Painful joint count	40 (69)
Swollen joint count	30 (51.7)
ESR (>28 mm/h)	34 (58.6)
CRP (>8 mg/L)	22 (37.9)
Rheumatoid factor (≥20 IU/mL)	34 (58.6)
Erosive arthritis	18 (31)

**Note:**

* Based on clinical expert’s diagnosis.

The clinical presentations of the RA patients included in this study is shown in Table 3. The most prevalent clinical presentations seen in the patients with RA were arthritis (51.7%), fatigue (46.6%), weight loss (44.8%), and loss of appetite (41.4%). While, the least commonly observed clinical presentations in the patients with RA were xerophthalmia (8.6%) and xerostomia (1.7%) (Table 3). Most of the RA patients (77.6%) simultaneously showed two or more of the clinical presentations. Lastly, on analyzing the co-morbid conditions of the patients with RA, it was observed that the majority of the patients with RA reported suffering from associated co-morbid conditions (Table 4), which include gastritis (50%), hypertension (44.8%), diabetes (55.2%), and allergies (34.5%). Whereas the least number of patients with RA had hypothyroidism (15.5%) and sexual compromise (1.7%).

**Table 3:** Clinical presentations of rheumatoid arthritis patients included in this study.

Clinical presentations	Rheumatoid arthritis* patients (*n* = 58) mean ± SD/frequency (percentage)
Arthritis	30 (51.7)
Fatigue	27 (46.6)
Weight loss	26 (44.8)
Loss of appetite	24 (41.4)
Fever	20 (34.5)
Arthralgia	11 (19.0)
Raynaud syndrome	10 (17.2)
Myalgias	9 (15.5)
Xerophthalmia	5 (8.6)
Xerostomia	1 (1.7)

**Note:**

* Based on clinical expert’s diagnosis.

**Table 4:** Co-morbidities in the rheumatoid arthritis patients.

Co-morbidities	Rheumatoid arthritis* patients (*n* = 58) Frequency (percentage)
Obesity	10 (17.2)
Gastritis	29 (50.0)
Hypertension	26 (44.8)
Diabetes	32 (55.2)
Sexual compromise	1 (1.7)
Allergy	20 (34.5)
Depression	10 (17.2)
Hypothyroidism	9 (15.5)

**Note:**

* Based on clinical expert’s diagnosis.

**Figure 1:**
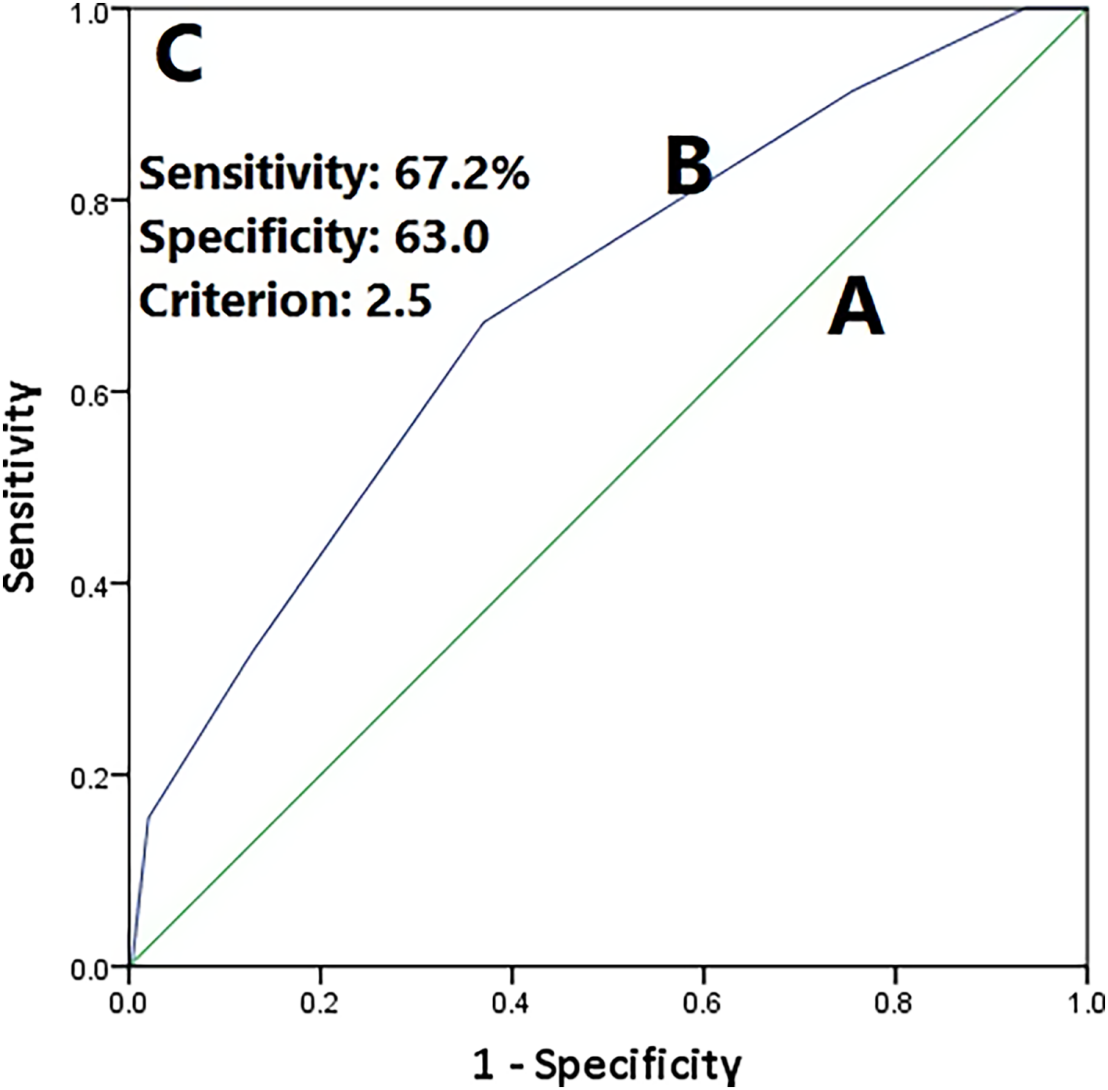
Receiver operating curve. (A) Line of no discrimination (AUC = 0.5), (B) prediction score based on the presence of six disease markers (painful joint count, swollen joint count, ESR (>28 mm/h), CRP level (>8 mg/L), rheumatoid Factor (≥20), and erosive arthritis) (AUC = 0.69), and (C) perfect test (AUC = 1.0). AUC, area under curve.

The diagnostic validity of the prediction score (based on presence of disease markers) was assessed by the ROC curve (Fig. 1). The sensitivity and specificity of the prediction score at the criterion score of 2.5 were 67.3% and 63.0%, respectively. The area under the curve was 0.69 (95% CI [0.62–0.76]). Cross tabulation of the classification based on the prediction score by the results of the clinical diagnosis is shown in Table S1.

## Discussion

Rheumatoid arthritis (RA) is a heterogeneous chronic autoimmune disorder that usually affects the joints, leading to joint damage with associated metabolic and psychological disturbances (Jones, Carley & Harrison, 2003; Kay et al., 2014). The joint swelling/joint damage in RA is the external reflection of the inflammation of synovial membrane following immune activation. The normal synovial compartment is intruded by leukocytes and the synovial fluid is overflowed with pro-inflammatory mediators that interact to generate an inflammatory cascade. This inflammatory cascade is characterized by the interactions of fibroblast-like synoviocytes (FLSs) with the cells of the innate immune system, including macrophages, monocytes, dendritic cells, mast cells, *etc.*, as well as with the cells of adaptive immune system such as B cells (humoral immunity) and T lymphocytes (cell-mediated immunity) (Kay & Upchurch, 2012). Early diagnosis of RA is generally difficult because its symptoms closely mimic those of the other diseases (Albishri, Alsabban & Altwairqi, 2015; Lubberts & van den Berg, 2003). Our study was conducted in the Madinah region of Saudi Arabia using a pool of 401 patients visiting the orthrpedic clinics of the three major hospitals (MGH, KFH and OH) of Madinah. The aim was to estimate the prevalence of RA among the patients visiting these orthropedic clinics and to study the clinical characteristic and the associated co-morbidities of the patients with RA. Diagnosis of RA traditionally depends upon physician- and patient-oriented views and responses to various symptoms and consequences of the disease. Physicians focus on the symptoms of the disease and assess the joint damage of the patients using various imaging techniques. They may also use the Health Assessment Questionnaire without Disability Index and the criteria from the American College of Rheumatology (ACR) and European League Against Rheumatism (EULAR) (Hosmer & Lemeshow, 2000; Houge et al., 2020; Lubberts & van den Berg, 2003; Luime et al., 2010; Lutf, Poil & Hammoudeh, 2014; McInnes & Schett, 2017; Michaud, Wallenstein & Wolfe, 2011; Mjaavatten & Bykerk, 2013). However, there are some limitations of the traditional disease activity measures, including reduced reliability, inconsistency, *etc.* (Mjaavatten & Bykerk, 2013). Furthermore, the results can be complicated by comorbidities, resulting in further uncertainty around the patient- and physician-reported assessments on RA disease activity (Myasoedova et al., 2010; Nielsen et al., 2012). Therefore, the single and multiple biomarker tests are equally important in the diagnosis and prognosis as well as treatment of RA (Nielsen et al., 2012; Nilsson et al., 2021; Padyukov et al., 2003; Prevoo et al., 1995; Raciborski et al., 2017; Rajapakse, 1987; Romeo & Fonseca, 2021). Keeping in view the above facts about the diagnosis and prognosis of RA, the diagnosis of the RA patients included in this study, as reported by the physician, was revealed from their medical records. Based upon the physician diagnosis, the prevalence of RA in patients visiting the orthropedic clinics of the selected hospitals of Madinah was found to be 14.46%. The epidemiological reports regarding RA in Saudi Arabia are suboptimal and the exact prevalence of RA in the Saudi population remains uncertain. However, there are few studies which have reported the prevalence of RA in some regions of Saudi Arabia. One of the studies, which was published in 1998 and has been carried out in Qassim region, reported the RA prevalence of 0.22% in that region (Heegaard et al., 2013). Another study conducted in the Taif region reported a RA prevalence of 0.3% in the study population (Ronnelid et al., 2005). However, the high prevalence of RA in our study compared to the earlier published studies may be explained based on the regional differences and differences in the study design. Our study population includes the patients suspecting of RA, bearing some RA symptoms and therefore specifically visiting the orthropedic clinics of the selected hospitals of Madinah region for diagnosis and treatment. Whereas, the above mentioned previous studies have included the general population as study subjects and their data is survey based as compared to clinical data.

In this study, it was observed that the mean age of patients with RA was 49.97 ± 18.34 years, which is in agreement with the well-established notion that RA mostly affects the middle aged and older aged people (Safiri et al., 2019). The vast majority of the patients with RA was represented by female gender (60.3%), which is in agreement with the known prediction of the disease (Safiri et al., 2019; Sanmartí, Ruiz-Esquide & Hernández, 2013). Our assessments showed that females are more prone to RA, and genetic and hormonal factors might contribute to sexual difference in the prevalence of RA (Saraux et al., 2001; Scott, Wolfe & Huizinga, 2010). RF was observed to be positive in 58.6% of patients with RA, resembling the results reported in other studies (Al-Ghamdi, 2009; Shmerling & Delbanco, 1991; Smolen et al., 2003). In this study, the most common disease marker of the RA patients was joint pain (69%), followed by swollen joints (51.7%) and erosive arthritis (31%). These observations were similar to those previously published in other studies (Al-Ghamdi, 2009; Smolen et al., 2003; Weyand et al., 1998; Yu et al., 2019).

The diagnostic validity of the prediction score generated based on the presence of six disease activity markers (painful joint count, swollen joint count, ESR (>28 mm/h), CRP level (>8 mg/L), RF (≥20), and erosive arthritis) was slightly lower than the acceptable range of 0.7 to 0.8 (Zhang et al., 2020). This is the first study to evaluate diagnostic applicability of a prediction score based on disease activity markers. Future studies may further build on this outcome by exploring methods to enhance performance of the prediction score. Some of the strategies that may be tested by future studies are (i) using ordinal or continuous forms of disease activity marker values instead of dichotomous form used in this study, (ii) develop a regression-based model, which could not be implemented in the present study because of data distribution, and sample size problems, and (iii) develop a prinicipal factor score based on the ordinal or continuous forms of disease activity marker values, which can be further used to predict clinical diagnosis of the RA.

We recognize several weaknesses in our study. Among the limitations of our study are the smaller sample size and the single region based data from only three selected hospitals. Therefore, additional multi-regional and nation wide studies on the prevalence of RA in Saudi Arabia are required for developing efficient management approaches for disease control. We have reported the prevalence of RA in patients visiting the orthopaedic clinics of the selected hospitals. Therefore, the results cannot be directly generalized to the general population of Madinah. In addition, the data regarding the treatment of the RA patients with or without reported comorbidities, and the treatment outcome thereof, was not collected/reported in this study. Furthermore, 74% of the study subjects were females, and adjustment for gender was not performed in the analysis. The 74% females in the sample is not representative of the general population in Madinah, therefore, the results cannot be directly generalized to the general population of Madinah.

## Conclusions

The prevalence of RA in patients visiting the orthropedic clinics of the selected hospitals of the Madinah region of Saudi Arabia was estimated to be 14.46%, which is higher than reported by some other studies conducted in different regions of Saudi Arabia. This variation is because of the differences in study design and due to the regional differences. Middle and older aged individuals were more prone to be affected by the disease. Further, the prevalence of RA was found to be higher in women as compared to men, which is well supported by the available literature. The most common disease marker of the RA patients was painful joint count, whereas, the most common clinical presentation and comorbidity of RA patients was arthritis and diabetes, respectively.

## Supplemental Information

10.7717/peerj.14362/supp-1 Supplemental Information 1 Cross tabulation of the index test results (or their distribution) by the results of the reference standard.

10.7717/peerj.14362/supp-2 Supplemental Information 2 Raw data.

10.7717/peerj.14362/supp-3 Supplemental Information 3 STANDARD Checklist.

## Additional Information and Declarations

### Data Availability

The following information was supplied regarding data availability: The raw data is available in the Supplemental Files.
