# Supplementary material for: Prevalence of rheumatoid arthritis and diagnostic validity of a prediction score, in patients visiting orthropedic clinics in the Madinah region of Saudi Arabia: a retrospective cross-sectional study
Source: PeerJ. 2022 Nov 15;10:e14362. doi: 10.7717/peerj.14362 (PMC9673770; doi:10.7717/peerj.14362)
Supplement: Supplemental Information 1 [file peerj-10-14362-s001.docx]

**Title: Prevalence of rheumatoid arthritis and diagnostic validity of a prediction score, in patients visiting orthropedic clinics in the Madinah region of Saudi Arabia: A retrospective cross-sectional study**

Shabir Ahmad Mir ^1,*^, Mamdooh Noor ^1^, Md Dilshad Manzar ^2^, Bader Alshehri ^1^, Mohammed Alaidarous ^1, 3^, Abdul Aziz Bin Dukhyil ^1^, Saeed Banawas ^1, 3, 4^, Yahya Madkhali ^1^, Sadaf Jahan ^1^, Faizan Z Kashoo ^5^, Danish Iqbal ^1^, Qamar Zia ^1, 3^, Suliman A Alsagaby^1^, Sahar ALDosari^1^

^1^ Department of Medical Laboratory Sciences, College of Applied Medical Science, Majmaah University, Al Majmaah-11952, Saudi Arabia.

^2^ Department of Nursing, College of Applied Medical Sciences, Majmaah University, Al Majmaah 11952, Saudi Arabia

^3^ Health and Basic Sciences Research Center, Majmaah University, Al Majmaah 11952, Saudi Arabia

^4^ Department of Biomedical Sciences, Oregon State University, Corvallis, OR 97331, USA

^5^ Department of Physical Therapy and Health Rehabilitation, College of Applied Medical Sciences, Majmaah University, Al Majmaah 11952, Saudi Arabia.

^*^ Corresponding author

Dr Shabir Ahmad Mir, PhD

Assistant Professor

Department of Medical Laboratory Sciences, College of Applied Medical Science, Majmaah University, Saudi Arabia.

Phone: +966536300645, Email: [s.mir@mu.edu.sa](mailto:s.mir@mu.edu.sa)

**Supplement table 1:** Cross tabulation of the index test results (or their distribution)
by the results of the reference standard

|  | | | | | |
| --- | --- | --- | --- | --- | --- |
|  | | | Classification based on the proposed cut-off prediction score | | Total |
|  |  |  | Non-rheumatic | Rheumatoid arthritis |  |
| clinical diagnosis | Non-rheumatic | Count | 216 | 127 | 343 |
|  |  | % within clinical diagnosis | 63.0% | 37.0% | 100.0% |
|  | Rheumatoid arthritis | Count | 19 | 39 | 58 |
|  |  | % within clinical diagnosis | 32.8% | 67.2% | 100.0% |
| Total | | Count | 235 | 166 | 401 |
|  |  | % within clinical diagnosis | 58.6% | 41.4% | 100.0% |

Index test results: Classification based on the proposed cut-off prediction score

Reference standard: clinical diagnosis
